# Supplementary material for: Rapid and reversible epigenome editing by endogenous chromatin regulators
Source: Nat Commun. 2017 Sep 15;8:560. doi: 10.1038/s41467-017-00644-y (PMC5601922; doi:10.1038/s41467-017-00644-y)
Supplement: Supplementary file 1 — Supplementary Information [file 41467_2017_644_MOESM1_ESM.pdf]

Description of Supplementary Files

Title: Supplementary Information

Description: Supplementary Figures, Supplementary Methods, and Supplementary Tables

Title: Peer Review File

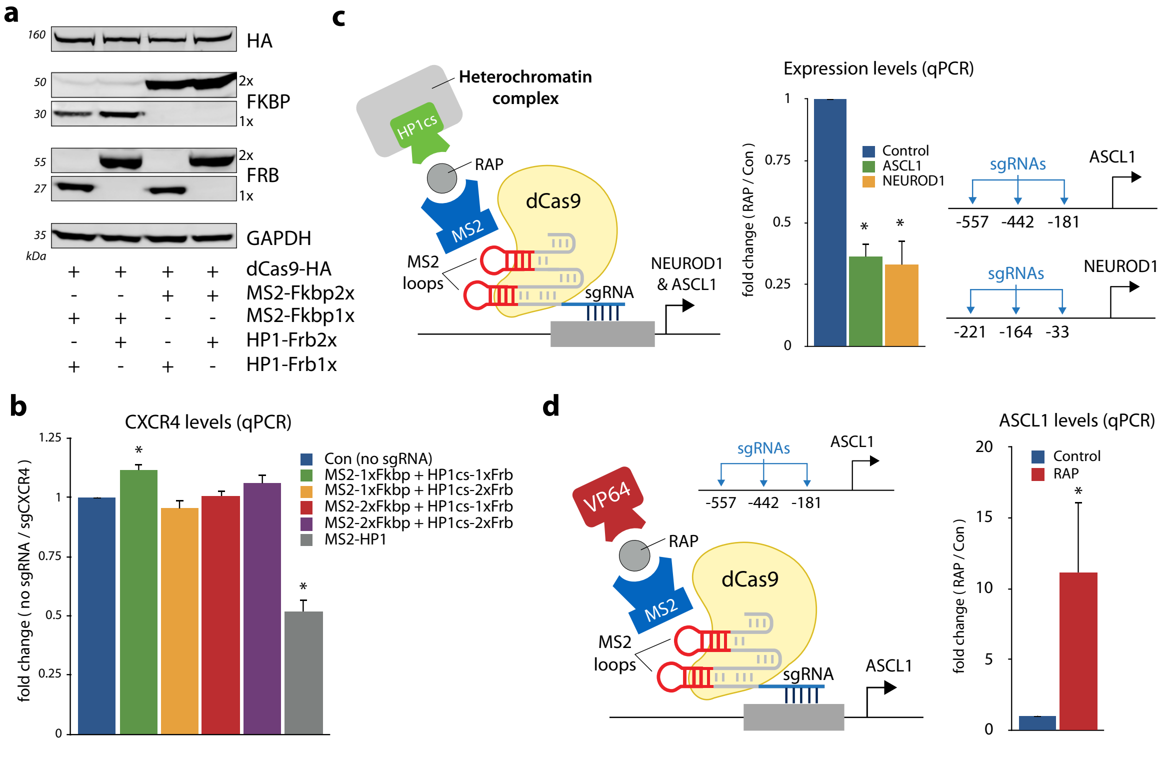

Supplementary Figure 1 | Inducible recruitment of HP1cs-Frb and VP64-Frb leads to silencing and activation of target gene expression. (**a**) Western blot analysis to determine protein overexpression levels in HEK 293 cells expressing dCas9-HA, MS2-Fkbp (1x or 2x) and HP1-Frb (1x or 2x). GAPDH levels measured for loading control. (**b**) CXCR4 expression levels measured by qPCR in dCas9 expressing HEK 293 cells with and without CXCR4 targeting sgRNAs for all four inducible conditions as well as the direct fusion. n=3; error bars s.e.m. $p < 0.05$. (**c**) Schematic representation of heterochromatin complex recruitment to the ASCL1 and NEUROD1 loci in HEK 293 cells. Ascl1 and Neurod1 expression levels are reduced after 5 days of RAP treatment to recruit HP1cs to respective loci. n=3; error bars s.e.m.; $p < 0.05$. (**d**) Schematic representation of inducible VP64 activator recruitment to the ASCL1 locus in HEK 293 cells. Ascl1 expression levels increase 10-fold after 48h of RAP treatment. n=3; error bars s.e.m.; $p < 0.05$.

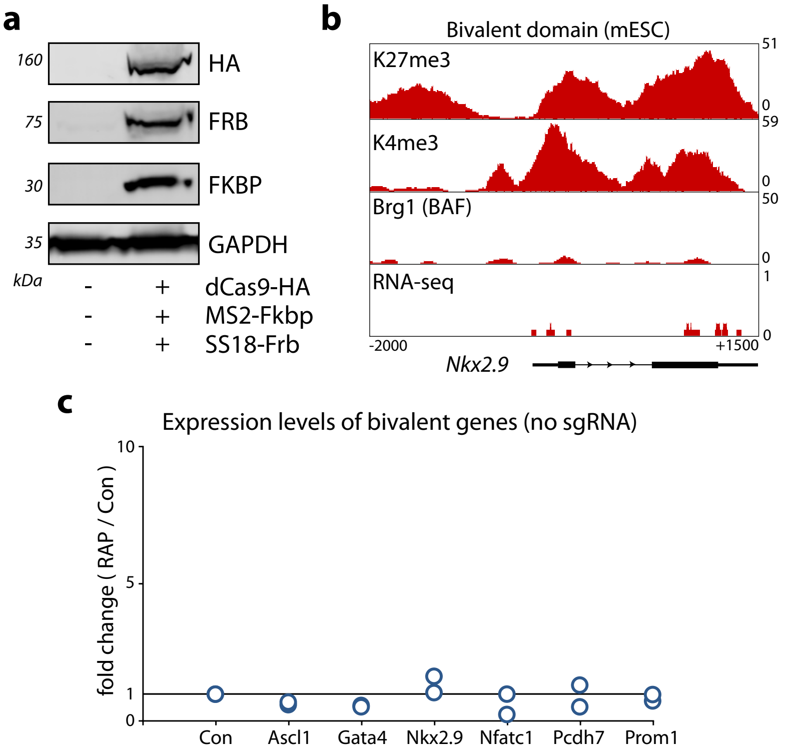

Supplementary Figure 2 | BAF complex recruitment to multiple bivalent loci in mESCs. (**a**) Western blot analysis to determine protein overexpression levels in mESCs expressing dCas9-HA, MS2-Fkbp and SS18-Frb. GAPDH levels measured for loading control. (**b**) The Nkx2.9 gene is bivalent in mESCs and expressed during neural progenitor differentiation into motor neurons. Published ChIP-seq datasets[30,36,37] show that this locus is characterized by high levels of H3K4me3 and H3K27me3, hallmarks of bivalency, as well as low levels of Brg1 (BAF) binding. Furthermore, Nkx2.9 is not expressed in mESCs, as shown by published RNA-seq datasets[38]. (**c**) No sgRNA control experiment to rule out RAP dependent induction of bivalent gene expression. After 48h RAP treatment in mESCs expressing dCas9-HA, MS2-Fkbp and SS18-Frb but no sgRNAs, no significant changes in gene expression levels were detected by qPCR. n=2 per condition.

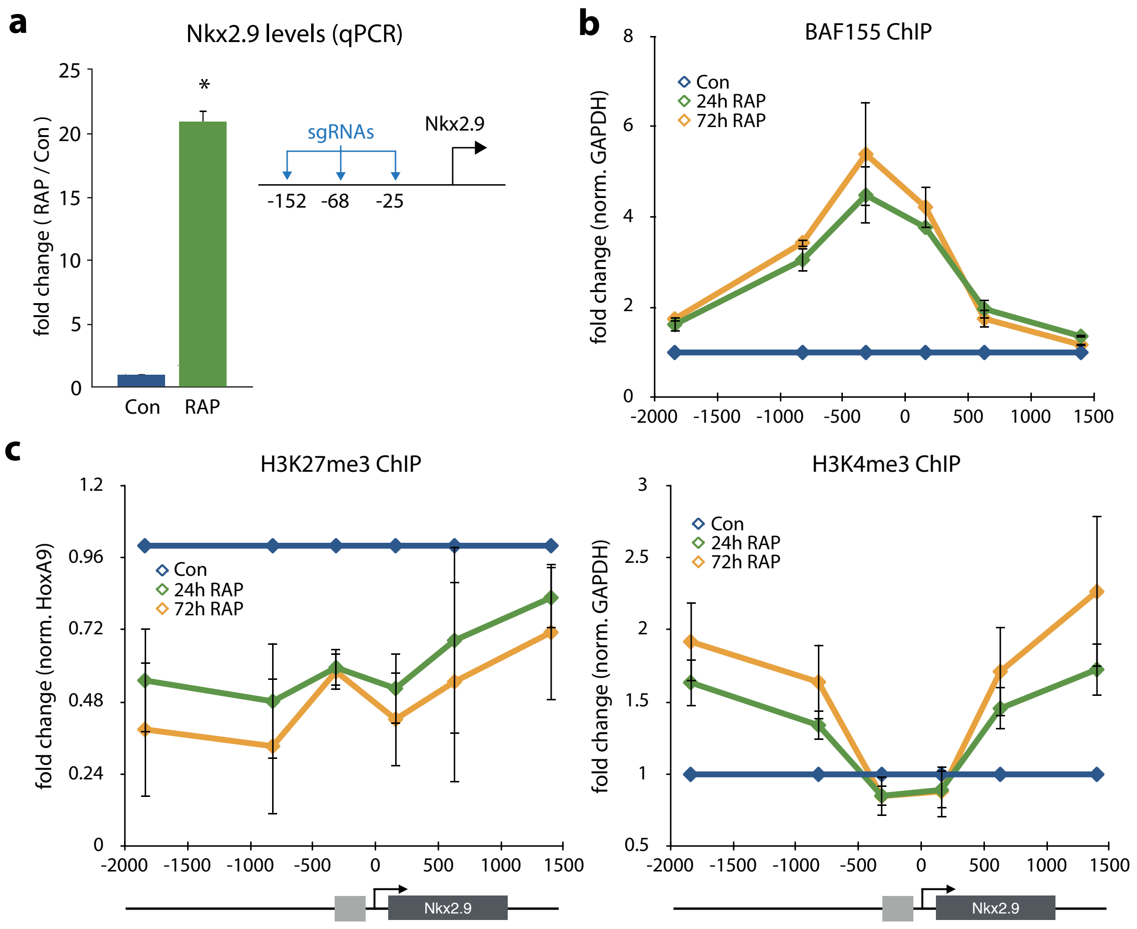

Supplementary Figure 3 | Extended time-course of BAF complex recruitment to the bivalent Nkx2.9 locus in mESCs. (**a**) After 48h RAP treatment to target the BAF complex to the Nkx2.9 locus, we detected a 20-fold increase in Nkx2.9 expression by qPCR. n=4; error bars s.e.m.; $p < 0.05$. (**b**) BAF155 ChIP shows recruitment of the intact BAF complex via the SS18-Frb subunit. We observed a 5-fold increase in BAF155 levels over the recruitment site after 24h and 72h of RAP treatment (-152bp, -68bp & -25bp from Nkx2.9 TSS). n=3; error bars s.e.m. (**c**) ChIP experiments show loss of H3K27me3 levels over the bivalent locus after 24h and 72h of RAP treatment. In contrast, H3K4me3 levels increase over the gene body as well as upstream of the recruitment site, suggesting bi-directional transcription after 24h and 72h of RAP treatment to recruit the BAF complex. n=3; error bars s.e.m. $p < 0.05$.

Gene	sgRNA1	sgRNA2	sgRNA3
CXCR4	-116<br>Fw: CAC CGG CAG ACG CGA GGA AGG AGG GCG C<br>Rv: AAA CGC GCC CTC CTT CCT CGC GTC TGC C	-162<br>Fw: CAC CGC CGA CCA CCC GCA AAC AGC A<br>Rv: AAA CTG CTG TTT GCG GGT GGT CGG C	-193<br>Fw: CAC CGG CCT CTG GGA GGT CCT GTC CGG CTC<br>Rv: AAA CGA GCC GGA CAG GAC CTC CCA GAG GCC
ASCL1	-181<br>Fw: CAC CGC GGG AGA AAG GAA CGG GAG G<br>Rv: AAA CCC TCC CGT TCC TTT CTC CCG C	-442<br>Fw: CAC CGT CCA ATT TCT AGG GTC ACC G<br>Rv: AAA CCG GTG ACC CTA GAA ATT GGA C	-557<br>Fw: CAC CGA AGA ACT TGA AGC AAA GCG C<br>Rv: AAA CGC GCT TTG CTT CAA GTT CTT C
NEUROD1	-221<br>Fw: CAC CGA GGT CCG CGG AGT CTC TAA C<br>Rv: AAA CGT TAG AGA CTC CGC GGA CCT C	-164<br>Fw: CAC CGA CCT GCC CAT TTG TAT GCC G<br>Rv: AAA CCG GCA TAC AAA TGG GCA GGT C	-33<br>Fw: CAC CGA GGG GAG CGG TTG TCG GAG G<br>Rv: AAA CCC TCC GAC AAC CGC TCC CCT C
Oct4	-239<br>Fw: CAC CGC GAT GGG GCA TCC GAG CAA C<br>Rv: AAA CGT TGC TCG GAT GCC CCA TCG C	-114<br>Fw: CAC CGA GAC GTC CCC AAC CTC CGT C<br>Rv: AAA CGA CGG AGG TTG GGG ACG TCT C	-396<br>Fw: CAC CGA TCT AGG ACT CTA GAC GGG T<br>Rv: AAA CAC CCG TCT AGA GTC CTA GAT C
Gata4	Fw: CAC CGG GGG CGG GGG AGC CCG GAC C<br>Rv: AAA CGG TCC GGG CTC CCC CGC CCC C	Fw: CAC CGG GGG CCC GGG GAA CCG CGC C<br>Rv: AAA CGG CGC GGT TCC CCG GGC CCC C	Fw: CAC CGG CAT GGA CTT TGC CTG TTG G<br>Rv: AAA CCC AAC AGG CAA AGT CCA TGC C
Nkx2.9	Fw: CAC CGG GGG CGG GTG CCG GGC GGG G<br>Rv: AAA CCC CCG CCC GGC ACC CGC CCC C	Fw: CAC CGG GGG CGG AGA TGG CAC CTT C<br>Rv: AAA CGA AGG TGC CAT CTC CGC CCC C	Fw: CAC CGA CCA AAG TGG GGA CAG ATA A<br>Rv: AAA CTT ATC TGT CCC CAC TTT GGT C
Nfatc1	Fw: CAC CGG CGG CCG GCG GGC GGG AGC C<br>Rv: AAA CGG CTC CCG CCC GCC GGC CGC C	Fw: CAC CGA AAA GGC AGA AGG CAG AGC C<br>Rv: AAA CGG CTC TGC CTT CTG CCT TTT C	Fw: CAC CGA CGC CCC TGC CCG CCC GAG C<br>Rv: AAA CGC TCG GGC GGG CAG GGG CGT C
Pcdh7	Fw: CAC CGA GAA GGA GGG AGG GAG GGG A<br>Rv: AAA CTC CCC TCC CTC CCT CCT TCT C	Fw: CAC CGT GGA GAG AGT GAG AGA GGG G<br>Rv: AAA CCC CCT CTC TCA CTC TCT CCA C	Fw: CAC CGT TGA TCC CTT TGC AGT TCA A<br>Rv: AAA CTT GAA CTG CAA AGG GAT CAA C
Prom1	Fw: CAC CGC CCG TCC CAC TGC TCC GCC C<br>Rv: AAA CGG GCG GAG CAG TGG GAC GGG C	Fw: CAC CGG GGT CTC TGG CAG AGG TTT G<br>Rv: AAA CCA AAC CTC TGC CAG AGA CCC C	Fw: CAC CGA CCA CGG GAC TCC AGG ACT C<br>Rv: AAA CGA GTC CTG GAG TCC CGT GGT C

Supplementary Table 1 | Primer sequences for the sgRNAs cloned into the Lentiviral vector: Lv U6 sgRNA-2xMS2-RNA EF1 Zeo.

Locus (bases from TSS)	Forward primer	Reverse primer
CXCR4 (-166)	GTT TGC GGG TGG TCG GTA	CCT GTC TTC AGG CGC ATC C
CXCR4 (+951)	CTA TCC CCG GAG CGC AAA T	TTC GAG AGT TTG GGG TCG TG
Oct4 (-739)	GCA CTT CTC TGG GGT CTC TG	ACC CAC CCG TCT AGA GTC CT
Oct4 (-130)	GTT TGT GAG GTG TCC GGT GA	GAC AAC CCT TAG GAC GGG AC
Oct4 (+254)	GTC CGC CCG CAT ACG AGT	TTG GGG CGG TCG GCA C
Nkx2.9 (-1840)	GCG GGA GGT GGT AGA GTT TT	AGG ATG CAC AGG TTT CCA GG
Nkx2.9 (-820)	CTC CAT TCG AGG ACC CAA GG	CTG CTA ACT GGC ACC GAC TT
Nkx2.9 (-315)	TCT TGG GTG GCG AAC AGT G	AAT AAA GTC GCT CCA CCC TCC
Nkx2.9 (+160)	CCG CTC CTA AGG ATG GAA GT	TTC AAA GCC CTC CGA GTA GC
Nkx2.9 (+630)	ATC CCG GTC TTT TCG GAT CG	TGC GTC TGA GTC CAC ACA TC
Nkx2.9 (+1400)	ACC TCT GCC GTT GTT GCT C	GCC TTC GGA TAT GGC AGC AT
Gapdh	CTC TGC TCC TCC CTG TTC C	TCC CTA GAC CCG TAC AGT GC
HoxA9	AAG AAG GAA AAG GGG AAT GG	TCA CCT CGC CTA GTT TCT GG

Supplementary Table 2 | Sequence information for the ChIP-qPCR primers used for the CXCR4, Oct4 and Nkx2.9 loci.

Gene	amplicon 1	amplicon 2
Gata4	Fw: CCA TCT CGC CTC CAG AGT<br>Rv: CTG GAA GAC ACC CCA ATC TC	Fw: CAG GCA TTG CAC AGG TAG TG<br>Rv: CTG GAA GAC ACC CCA ATC TC
Nkx2.9	Fw: CTC GTC CGA GGA CAG GTA GT<br>Rv: TTA CCT GAA CAG GAT GCG AA	Fw: GAG TCT GCA GGG CTT GTC TC<br>Rv: TTA CCT GAA CAG GAT GCG AA
Nfatc1	Fw: CAT AGT GAG CCC TGT GGT GA<br>Rv: AGT CTC TTT CCC CGA CAT CA	Fw: TCA TAG TGA GCC CTG TGG TG<br>Rv: GAA GCC CAA GTC TCT TTC CC
Pcdh7	Fw: GAA TTC AGC CAA ACA CCG TAA<br>Rv: GAT CAC TGC TCC GAG TAC AGC	Fw: TTC AGC CAA ACA CCG TAA TG<br>Rv: GTG GGA GCA GGA GAC AAC AT
Prom1	Fw: TAG AGG GAA GTC ATT CGG CT<br>Rv: CCC AAG ATA CCT TCA ATG CTG	Fw: GGC AAT CTC CTT GGA ATC AA<br>Rv: CAA AAT GGT GCA CAT CTT CCT
Gapdh	Fw: AGG TCG GTG TGA ACG GAT TTG<br>Rv: TGT AGA CCA TGT AGT TGA GGT CA	
GAPDH	Fw: GCC AGC CGA GCC ACA T<br>Rv: CTT TAC CAG AGT TAA AAG CAG CCC	
ACTINB	Fw: CAT GTA CGT TGC TAT CCA GGC<br>Rv: CTC CTT AAT GTC ACG CAC GAT	
CXCR4	Fw: ACT ACA CCG AGG AAA TGG GCT<br>Rv: CCC ACA ATG CCA GTT AAG AAG A	
ASCL1	Fw: CGC GGC CAA CAA GAA GAT G<br>Rv: CGA CGA GTA GGA TGA GAC CG	
NEUROD1	Fw: GGA TGA CGA TCA AAA GCC CAA<br>Rv: GCG TCT TAG AAT AGC AAG GCA	

Supplementary Table 3 | Sequence information for the RT-qPCR primers used in this study.

Supplementary Note 1 | Plasmid sequence information for the lentiviral constructs used in this study:

Lv EF1a MS2-Fkbp(1x or 2x) 2A *Hygro*
Lv EF1a MS2-HP1cs 2A *Hygro*
Lv EF1a dCas9-HA 2A *Blast*
Lv EF1a HP1cs-Frb(1x or 2x) PGK *Puro*
Lv EF1a SS18-Frb PGK *Puro*
Lv U6 sgRNA-MS2-RNA EF1 *Zeo*

These plasmids and the corresponding complete sequence information have been deposited to Addgene. The plasmids used are modified lentiviral constructs obtained from Addgene (#61425, #61426, #61427 and #44195). The lentiviral backbones, promoters and resistance marker sequences were unmodified. The detailed sequence information for the fusion proteins cloned for this study are listed below:

MS2-NLS-FKBP sequence (sub-cloned into Addgene #61426):

ATGGCTTCAAACTTTACTCAGTTCGTGCTCGTGGACAATGGTGGGACAGGGGATGTGACAGTG
GCTCCTTCTAATTTCGCTAATGGGGTGGCAGAGTGGATCAGCTCCAACTCACGGAGCCAGGCC
TACAAGGTGACATGCAGCGTCAGGCAGTCTAGTGCCCAGAAGAGAAAGTATACCATCAAGGTG
GAGGTCCCCAAAGTGGCTACCCAGACAGTGGGCGGAGTCGAACTGCCTGTCGCCGCTTGGAG
GTCCTACCTGAACATGGAGCTCACTATCCCAATTTTCGCTACCAATTCTGACTGTGAACTCATC
GTGAAGGCAATGCAGGGGCTCCTCAAAGACGGTAATCCTATCCCTTCCGCCATCGCCGCTAAC
TCAGGTATCTACAGCGCTGGAGGAGGTGGAAGCGGAGGAGGAGGAAGCGGAGGAGGAGGTA
GCGGACCTAAGAAAAAGAGGAAGGTGGCGGCCGCTGGATCCGGCGTGCAGGTGGAGACTAT
CTCCCCAGGGGACGGGCGCACCTTCCCCAAGCGCGGCCAGACCTGCGTGGTGCACTACACC
GGGATGCTTGAAGATGGAAAGAAATTTGATTCCTCCCGGGACAGAAACAAGCCCTTTAAGTTT
ATGCTAGGCAAGCAGGAGGTGATCCGAGGCTGGGAAGAAGGGGTTGCCCAGATGAGTGTGG
GTCAGAGAGCCAAACTGACTATATCTCCAGATTATGCCTATGGTGCCACTGGGCACCCAGGCA
TCATCCCACCACATGCCACTCTCGTCTTCGATGTGGAGCTTCTAAAACTGGAA

MS2-NLS-FKBP-FKBP sequence (sub-cloned into Addgene #61426):

ATGGCTTCAAACTTTACTCAGTTCGTGCTCGTGGACAATGGTGGGACAGGGGATGTGACAGTG
GCTCCTTCTAATTTCGCTAATGGGGTGGCAGAGTGGATCAGCTCCAACTCACGGAGCCAGGCC
TACAAGGTGACATGCAGCGTCAGGCAGTCTAGTGCCCAGAAGAGAAAGTATACCATCAAGGTG
GAGGTCCCCAAAGTGGCTACCCAGACAGTGGGCGGAGTCGAACTGCCTGTCGCCGCTTGGAG
GTCCTACCTGAACATGGAGCTCACTATCCCAATTTTCGCTACCAATTCTGACTGTGAACTCATC
GTGAAGGCAATGCAGGGGCTCCTCAAAGACGGTAATCCTATCCCTTCCGCCATCGCCGCTAAC
TCAGGTATCTACAGCGCTGGAGGAGGTGGAAGCGGAGGAGGAGGAAGCGGAGGAGGAGGTA
GCGGACCTAAGAAAAAGAGGAAGGTGGCGGCCGCTGGATCCGGTGTACAGGTTGAGACGATC
TCGCCTGGGGACGGCCGCACTTTCCCGAAACGCGGTCAAACTTGTGTAGTACACTACACAGG
AATGCTCGAGGACGGGAAAAAGTTTGACAGTTCACGCGATCGCAATAAGCCATTCAAGTTTAT
GCTCGGAAAGCAGGAGGTAATACGCGGGTGGGAAGAGGGAGTCGCACAGATGTCTGTAGGC
CAACGTGCTAAGTTAACGATTTCCCCGGATTACGCATATGGGGCGACGGGTCACCCGGGTATA
ATTCCGCCTCATGCTACCTTAGTTTTCGACGTAGAATTACTCAAACTTGAAGGAGGTGGCTCCG
GGGGAGGGAGTGGATCCGGCGTGCAGGTGGAGACTATCTCCCCAGGGGACGGGCGCACCTT
CCCCAAGCGCGGCCAGACCTGCGTGGTGCACTACACCGGGATGCTTGAAGATGGAAAGAAAT
TTGATTCCTCCCGGGACAGAAACAAGCCCTTTAAGTTTATGCTAGGCAAGCAGGAGGTGATCC

GAGGCTGGGAAGAAGGGGTTGCCCAGATGAGTGTGGGTCAGAGAGCCAAACTGACTATATCT
CCAGATTATGCCTATGGTGCCACTGGGCACCCAGGCATCATCCCACCACATGCCACTCTCGTC
TTCGATGTGGAGCTTCTAAAACTGGAATGTACA

HP1-Frb (sub-cloned into #44195):

ATGAAGGAGGGTGAAAACAATAAGCCCAGGGAGAAATCAGAAGGAAACAAGAGGAAATCCAG
TTTCTCCAACAGCGCTGATGATATTAAATCTAAAAAAAAGAGAGAGCAAAGCAATGATATCGCT
CGGGGCTTTGAGAGAGGACTGGAACCAGAAAAGATCATCGGAGCAACAGATTCCTGCGGTGA
CTTAATGTTCTTAATGAAATGGAAAGACACAGATGAAGCTGACCTGGTTCTTGCAAAAGAAGCT
AACGTGAAGTGTCCACAGATTGTGATAGCATTTTATGAAGAGAGACTGACGTGGCACGCATAT
CCAGAGGATGCGGAAAACAAAGAAAAAGAAAGCGCGAAGAGCAAGGGCGAATTCGACCCAGC
TTTCTTGTACAAAGTGGTTGATCTAGAGGGCCCTATGTGGCATGAAGGCCTGGAAGAGGCATC
TCGTTTGTACTTTGGGGAAAGGAACGTGAAAGGCATGTTTGAGGTGCTGGAGCCCTTGCATGC
TATGATGGAACGGGGCCCCCAGACTCTGAAGGAAACATCCTTTAATCAGGCCTATGGTCGAGA
TTTAATGGAGGCCCAAGAGTGGTGCAGGAAGTACATGAAATCAGGGAATGTCAAGGACCTCAC
CCAAGCCTGGGACCTCTATTATCATGTGTTCCGACGAATCTCAAAGCAGTTCGGCTAG

HP1-Frb-Frb-V5 (modified from Addgene #44195):

ATGAAGGAGGGTGAAAACAATAAGCCCAGGGAGAAATCAGAAGGAAACAAGAGGAAATCCAG
TTTCTCCAACAGCGCTGATGATATTAAATCTAAAAAAAAGAGAGAGCAAAGCAATGATATCGCT
CGGGGCTTTGAGAGAGGACTGGAACCAGAAAAGATCATCGGAGCAACAGATTCCTGCGGTGA
CTTAATGTTCTTAATGAAATGGAAAGACACAGATGAAGCTGACCTGGTTCTTGCAAAAGAAGCT
AACGTGAAGTGTCCACAGATTGTGATAGCATTTTATGAAGAGAGACTGACGTGGCACGCATAT
CCAGAGGATGCGGAAAACAAAGAAAAAGAAAGCGCGAAGAGCAAGGGCGAATTCGACCCAGC
TTTCTTGTACAAAGTGGTTGATCTAGAGGGCCCTATGTGGCATGAAGGCCTGGAAGAGGCATC
TCGTTTGTACTTTGGGGAAAGGAACGTGAAAGGCATGTTTGAGGTGCTGGAGCCCTTGCATGC
TATGATGGAACGGGGCCCCCAGACTCTGAAGGAAACATCCTTTAATCAGGCCTATGGTCGAGA
TTTAATGGAGGCCCAAGAGTGGTGCAGGAAGTACATGAAATCAGGGAATGTCAAGGACCTCAC
CCAAGCCTGGGACCTCTATTATCATGTGTTCCGACGAATCTCAAAGCAGTTCGGCGGCGGAGG
TAGCGGCGGTGGATCAATGTGGCACGAGGGATTGGAGGAAGCTAGCAGACTTTATTTCGGTG
AGCGCAATGTCAAGGGAATGTTCGAGGTCCTCGAACCTCTCCACGCCATGATGGAGAGAGGA
CCTCAAACCCTCAAAGAGACTAGTTTCAACCAAGCTTACGGCAGGGACCTGATGGAAGCTCAG
GAATGGTGTCGCAAATATATGAAGAGCGGCAACGTGAAAGATTTGACACAGGCTTGGGATTTG
TACTACCACGTCTTTAGGAGGATTAGCAAACAATTCGAAGGTAAGCCTATCCCTAACCCTCTCC
TCGGTCTCGATTCTACGCGTACCGGTTAG

MS2-NLS-HP1 (sub-cloned into #61426)

ATGGCTTCAAACTTTACTCAGTTCGTGCTCGTGGACAATGGTGGGACAGGGGATGTGACAGTG
GCTCCTTCTAATTTCGCTAATGGGGTGGCAGAGTGGATCAGCTCCAACTCACGGAGCCAGGCC
TACAAGGTGACATGCAGCGTCAGGCAGTCTAGTGCCCAGAAGAGAAAGTATACCATCAAGGTG
GAGGTCCCCAAAGTGGCTACCCAGACAGTGGGCGGAGTCGAACTGCCTGTCGCCGCTTGGAG
GTCCTACCTGAACATGGAGCTCACTATCCCAATTTTCGCTACCAATTCTGACTGTGAACTCATC
GTGAAGGCAATGCAGGGGCTCCTCAAAGACGGTAATCCTATCCCTTCCGCCATCGCCGCTAAC
TCAGGTATCTACAGCGCTGGAGGAGGTGGAAGCGGAGGAGGAGGAAGCGGAGGAGGAGGTA
GCGGACCTAAGAAAAAGAGGAAGGTGGCGGCCGCTGGATCCATGAAGGAGGGTGAAAACAAT
AAGCCCAGGGAGAAATCAGAAGGAAACAAGAGGAAATCCAGTTTCTCCAACAGCGCTGATGAT

ATTAAATCTAAAAAAAAGAGAGAGCAAAGCAATGATATCGCTCGGGGCTTTGAGAGAGGACTG
GAACCAGAAAAGATCATCGGAGCAACAGATTCCTGCGGTGACTTAATGTTCTTAATGAAATGGA
AAGACACAGATGAAGCTGACCTGGTTCTTGCAAAAGAAGCTAACGTGAAGTGTCCACAGATTG
TGATAGCATTTTATGAAGAGAGACTGACGTGGCACGCATATCCAGAGGATGCGGAAAACAAAG
AAAAAGAAAGCGCGAAGAGCTGTACA

Frb-Frb-V5-SS18 (sub-cloned into Addgene #44195):

ATGTGGCATGAAGGCCTGGAAGAGGCATCTCGTTTGTACTTTGGGGAAAGGAACGTGAAAGG
CATGTTTGAGGTGCTGGAGCCCTTGCATGCTATGATGGAACGGGGCCCCCAGACTCTGAAGG
AAACATCCTTTAATCAGGCCTATGGTCGAGATTTAATGGAGGCCCAAGAGTGGTGCAGGAAGT
ACATGAAATCAGGGAATGTCAAGGACCTCACCCAAGCCTGGGACCTCTATTATCATGTGTTCC
GACGAATCTCAAAGCAGTTCGGCGGCGGAGGTAGCGGCGGTGGATCAATGTGGCACGAGGG
ATTGGAGGAAGCTAGCAGACTTTATTTCGGTGAGCGCAATGTCAAGGGAATGTTCGAGGTCCT
CGAACCTCTCCACGCCATGATGGAGAGAGGACCTCAAACCCTCAAAGAGACTAGTTTCAACCA
AGCTTATGGCAGGGACCTGATGGAAGCTCAGGAATGGTGTCGCAAATATATGAAGAGCGGCA
ACGTGAAAGATTTGACACAGGCTTGGGATTTGTACTACCACGTCTTTAGGAGGATTAGCAAACA
ATTCGAAGGTAAGCCTATCCCTAACCCTCTCCTCGGTCTCGATTCTACGGGAGGCGGATCAGG
TGGCGGTAGCGCGGCCGCAATGTCTGTGGCGTTCGCAGCCCCGAGGCAGCGGGGCAAGGGC
GAAATCACGCCCGCCGCCATCCAGAAGATGCTGGATGAAAACAACCATCTTATTCAGTGTATA
ATGGACTATCAGAACAAAGGGAAGGCCTCGGAGTGCTCGCAGTATCAGCAGATATTGCATACA
AACCTGGTATACCTTGCTACAATAGCAGACTCTAATCAAAATATGCAGTCTCTCTTACCAGCAC
CGCCCACACAGACTATGCCAATGGGTCCTGGAGGGATGAGTCAGAGTGGCCCTCCACCCCCT
CCCCGCTCTCACAACATGCCTTCAGATGGAATGGTGGGTGGGGGCCCTCCTGCACCACACAT
GCAGAACCAGATGAACGGCCAGATGCCTGGGCCTAACCATATGCCAATGCAGGGACCTGGAC
CCAGTCAGCTCAGCATGACAAACAGCTCCATGAATATGCCTTCAAGTAGCCATGGCTCCATGG
GAGGTTACAACCATTCTGTGCCGTCATCCCAGAGCATGCCCGTGCAGAACCAGATGACAATGA
GTCAGGGGCAGCCAATGGGAAACTATGGTCCCAGACCAAACATGAATATGCAACCAAATCAAG
GGCCGATGATGCACCAGCAGCCTCCTTCTCAGCAGTACAATATGCCACCTGGAGGGGCACAG
CATTACCAAGGACAGCAGGCGCCCATGGGGCTGATGGGCCAAGTTAACCAAGGCAGTCACAT
GATGGGCCAGCGACAGATGCCTCCCTACAGACCTCCGCAACAGGGCCCACCACAGCAGTACT
CAGGCCAGGAAGACTATTATGGGGACCAATACAGTCATGGTGGACAAGGTCCTCCAGAAGGC
ATGAACCAGCAATATTACCCTGATGGTCATAATGATTACGGTTATCAGCAACCGTCGTATCCTG
AACAAGGCTACGATAGGCCTTATGAGGATTCCTCACAACATTACTACGAAGGAGGAAACTCCC
AGTATGGCCAACAGCAAGACGCTTACCAGGGACCACCTCCACAGCAAGGATACCCACCCCAG
CAGCAGCAGTACCCGGGACAGCAGGGATACCCAGGGCAGCAGCAGAGCTATGGTCCTTCGCA
GGGCGGTCCAGGTCCTCAGTATCCTAATTATCCTCAGGGTCAAGGTCAGCAGTATGGGGGCTA
TAGACCAACACAGCCAGGACCACCCCAGCCACCCCAGCAGAGGCCTTATGGGTACGACCAGG
GACAGTATGGAAATTACCAGCAGTGA

dCas9-NLS-HA sequence (sub-cloned into Addgene #61425):

ATGAAAAGGCCGGCGGCCACGAAAAAGGCCGGCCAGGCAAAAAAGAAAAAGGACAAGAAGTA
CAGCATCGGCCTGGCCATCGGCACCAACTCTGTGGGCTGGGCCGTGATCACCGACGAGTACA
AGGTGCCCAGCAAGAAATTCAAGGTGCTGGGCAACACCGACCGGCACAGCATCAAGAAGAAC
CTGATCGGAGCCCTGCTGTTCGACAGCGGCGAAACAGCCGAGGCCACCCGGCTGAAGAGAA
CCGCCAGAAGAAGATACACCAGACGGAAGAACCGGATCTGCTATCTGCAAGAGATCTTCAGCA

ACGAGATGGCCAAGGTGGACGACAGCTTCTTCCACAGACTGGAAGAGTCCTTCCTGGTGGAA
GAGGATAAGAAGCACGAGCGGCACCCCATCTTCGGCAACATCGTGGACGAGGTGGCCTACCA
CGAGAAGTACCCCACCATCTACCACCTGAGAAAGAAACTGGTGGACAGCACCGACAAGGCCG
ACCTGCGGCTGATCTATCTGGCCCTGGCCCACATGATCAAGTTCCGGGGCCACTTCCTGATCG
AGGGCGACCTGAACCCCGACAACAGCGACGTGGACAAGCTGTTCATCCAGCTGGTGCAGACC
TACAACCAGCTGTTCGAGGAAAACCCCATCAACGCCAGCGGCGTGGACGCCAAGGCCATCCT
GTCTGCCAGACTGAGCAAGAGCAGACGGCTGGAAAATCTGATCGCCCAGCTGCCCGGCGAGA
AGAAGAATGGCCTGTTCGGCAACCTGATTGCCCTGAGCCTGGGCCTGACCCCCAACTTCAAGA
GCAACTTCGACCTGGCCGAGGATGCCAAACTGCAGCTGAGCAAGGACACCTACGACGACGAC
CTGGACAACCTGCTGGCCCAGATCGGCGACCAGTACGCCGACCTGTTTCTGGCCGCCAAGAA
CCTGTCCGACGCCATCCTGCTGAGCGACATCCTGAGAGTGAACACCGAGATCACCAAGGCCC
CCCTGAGCGCCTCTATGATCAAGAGATACGACGAGCACCACCAGGACCTGACCCTGCTGAAA
GCTCTCGTGCGGCAGCAGCTGCCTGAGAAGTACAAAGAGATTTTCTTCGACCAGAGCAAGAAC
GGCTACGCCGGCTACATTGACGGCGGAGCCAGCCAGGAAGAGTTCTACAAGTTCATCAAGCC
CATCCTGGAAAAGATGGACGGCACCGAGGAACTGCTCGTGAAGCTGAACAGAGAGGACCTGC
TGCGGAAGCAGCGGACCTTCGACAACGGCAGCATCCCCCACCAGATCCACCTGGGAGAGCTG
CACGCCATTCTGCGGCGGCAGGAAGATTTTTACCCATTCCTGAAGGACAACCGGGAAAAGATC
GAGAAGATCCTGACCTTCCGCATCCCCTACTACGTGGGCCCTCTGGCCAGGGGAAACAGCAG
ATTCGCCTGGATGACCAGAAAGAGCGAGGAAACCATCACCCCCTGGAACTTCGAGGAAGTGG
TGGACAAGGGCGCTTCCGCCCAGAGCTTCATCGAGCGGATGACCAACTTCGATAAGAACCTG
CCCAACGAGAAGGTGCTGCCCAAGCACAGCCTGCTGTACGAGTACTTCACCGTGTATAACGA
GCTGACCAAAGTGAAATACGTGACCGAGGGAATGAGAAAGCCCGCCTTCCTGAGCGGCGAGC
AGAAAAAGGCCATCGTGGACCTGCTGTTCAAGACCAACCGGAAAGTGACCGTGAAGCAGCTG
AAAGAGGACTACTTCAAGAAAATCGAGTGCTTCGACTCCGTGGAAATCTCCGGCGTGGAAGAT
CGGTTCAACGCCTCCCTGGGCACATACCACGATCTGCTGAAAATTATCAAGGACAAGGACTTC
CTGGACAATGAGGAAAACGAGGACATTCTGGAAGATATCGTGCTGACCCTGACACTGTTTGAG
GACAGAGAGATGATCGAGGAACGGCTGAAAACCTATGCCCACCTGTTCGACGACAAAGTGAT
GAAGCAGCTGAAGCGGCGGAGATACACCGGCTGGGGCAGGCTGAGCCGGAAGCTGATCAAC
GGCATCCGGGACAAGCAGTCCGGCAAGACAATCCTGGATTTCCTGAAGTCCGACGGCTTCGC
CAACAGAAACTTCATGCAGCTGATCCACGACGACAGCCTGACCTTTAAAGAGGACATCCAGAA
AGCCCAGGTGTCCGGCCAGGGCGATAGCCTGCACGAGCACATTGCCAATCTGGCCGGCAGC
CCCGCCATTAAGAAGGGCATCCTGCAGACAGTGAAGGTGGTGGACGAGCTCGTGAAAGTGAT
GGGCCGGCACAAGCCCGAGAACATCGTGATCGAAATGGCCAGAGAGAACCAGACCACCCAGA
AGGGACAGAAGAACAGCCGCGAGAGAATGAAGCGGATCGAAGAGGGCATCAAAGAGCTGGG
CAGCCAGATCCTGAAAGAACACCCCGTGGAAAACACCCAGCTGCAGAACGAGAAGCTGTACC
TGTACTACCTGCAGAATGGGCGGGATATGTACGTGGACCAGGAACTGGACATCAACCGGCTG
TCCGACTACGATGTGGACCACATCGTGCCTCAGAGCTTTCTGAAGGACGACTCCATCGACAAC
AAGGTGCTGACCAGAAGCGACAAGGCCCGGGGCAAGAGCGACAACGTGCCCTCCGAAGAGG
TCGTGAAGAAGATGAAGAACTACTGGCGGCAGCTGCTGAACGCCAAGCTGATTACCCAGAGA
AAGTTCGACAATCTGACCAAGGCCGAGAGAGGCGGCCTGAGCGAACTGGATAAGGCCGGCTT
CATCAAGAGACAGCTGGTGGAAACCCGGCAGATCACAAAGCACGTGGCACAGATCCTGGACT
CCCGGATGAACACTAAGTACGACGAGAATGACAAGCTGATCCGGGAAGTGAAAGTGATCACC
CTGAAGTCCAAGCTGGTGTCCGATTTCCGGAAGGATTTCCAGTTTTACAAAGTGCGCGAGATC
AACAACTACCACCACGCCCACGACGCCTACCTGAACGCCGTCGTGGGAACCGCCCTGATCAA
AAAGTACCCTAAGCTGGAAAGCGAGTTCGTGTACGGCGACTACAAGGTGTACGACGTGCGGA
AGATGATCGCCAAGAGCGAGCAGGAAATCGGCAAGGCTACCGCCAAGTACTTCTTCTACAGCA
ACATCATGAACTTTTTCAAGACCGAGATTACCCTGGCCAACGGCGAGATCCGGAAGCGGCCTC

TGATCGAGACAAACGGCGAAACCGGGGAGATCGTGTGGGATAAGGGCCGGGATTTTGCCACC
GTGCGGAAAGTGCTGAGCATGCCCCAAGTGAATATCGTGAAAAAGACCGAGGTGCAGACAGG
CGGCTTCAGCAAAGAGTCTATCCTGCCCAAGAGGAACAGCGATAAGCTGATCGCCAGAAAGAA
GGACTGGGACCCTAAGAAGTACGGCGGCTTCGACAGCCCCACCGTGGCCTATTCTGTGCTGG
TGGTGGCCAAAGTGGAAAAGGGCAAGTCCAAGAAACTGAAGAGTGTGAAAGAGCTGCTGGGG
ATCACCATCATGGAAAGAAGCAGCTTCGAGAAGAATCCCATCGACTTTCTGGAAGCCAAGGGC
TACAAAGAAGTGAAAAAGGACCTGATCATCAAGCTGCCTAAGTACTCCCTGTTCGAGCTGGAA
AACGGCCGGAAGAGAATGCTGGCCTCTGCCGGCGAACTGCAGAAGGGAAACGAACTGGCCCT
GCCCTCCAAATATGTGAACTTCCTGTACCTGGCCAGCCACTATGAGAAGCTGAAGGGCTCCCC
CGAGGATAATGAGCAGAAACAGCTGTTTGTGGAACAGCACAAGCACTACCTGGACGAGATCAT
CGAGCAGATCAGCGAGTTCTCCAAGAGAGTGATCCTGGCCGACGCTAATCTGGACAAAGTGC
TGTCCGCCTACAACAAGCACCGGGATAAGCCCATCAGAGAGCAGGCCGAGAATATCATCCAC
CTGTTTACCCTGACCAATCTGGGAGCCCCTGCCGCCTTCAAGTACTTTGACACCACCATCGAC
CGGAAGAGGTACACCAGCACCAAAGAGGTGCTGGACGCCACCCTGATCCACCAGAGCATCAC
CGGCCTGTACGAGACACGGATCGACCTGTCTCAGCTGGGAGGCGACAGCGCTGGAGGAGGT
GGAAGCGGAGGAGGAGGAAGCGGAGGAGGAGGTAGCGGACCTAAGAAAAAGAGGAAGGTGG
CGGCCGCTGGATCCTATCCGTACGACGTACCAGACTACGCA

sgRNA-MS2 sequence : Complete sequence available at www.addgene.org/61427
